# Genome Features of *Asaia* sp. W12 Isolated from the Mosquito *Anopheles stephensi* Reveal Symbiotic Traits

**DOI:** 10.3390/genes12050752

**Published:** 2021-05-17

**Authors:** Shicheng Chen, Ting Yu, Nicolas Terrapon, Bernard Henrissat, Edward D. Walker

**Affiliations:** 1 Department of Clinical and Diagnostic Sciences, School of Health Sciences, Oakland University, 433 Meadowbrook Road, Rochester, MI 48309, USA; 2 Agro-Biological Gene Research Center, Guangdong Academy of Agricultural Sciences, Guangzhou 510640, China; yuting@agrogene.ac.cn; 3 Architecture et Fonction des Macromolécules Biologiques, Centre National de la Recherche Scientifique (CNRS), Aix-Marseille Université (AMU), UMR 7257, 13288 Marseille, France; nicolas.terrapon@univ-amu.fr (N.T.); Bernard.Henrissat@gmail.com (B.H.); 4 Institut National de la Recherche Agronomique (INRA), USC AFMB, 1408 Marseille, France; 5 Department of Biological Sciences, King Abdulaziz University, Jeddah 21412, Saudi Arabia; 6 Department of Microbiology and Molecular Genetics, Michigan State University, East Lansing, MI 48824, USA; walker@msu.edu

**Keywords:** *Asaia*, paratransgenesis, symbiotic traits, *Anopheles stephensi*, genome features

## Abstract

*Asaia* bacteria commonly comprise part of the microbiome of many mosquito species in the genera *Anopheles* and *Aedes*, including important vectors of infectious agents. Their close association with multiple organs and tissues of their mosquito hosts enhances the potential for paratransgenesis for the delivery of antimalaria or antivirus effectors. The molecular mechanisms involved in the interactions between *Asaia* and mosquito hosts, as well as *Asaia* and other bacterial members of the mosquito microbiome, remain underexplored. Here, we determined the genome sequence of *Asaia* strain W12 isolated from *Anopheles stephensi* mosquitoes, compared it to other *Asaia* species associated with plants or insects, and investigated the properties of the bacteria relevant to their symbiosis with mosquitoes. The assembled genome of strain W12 had a size of 3.94 MB, the largest among *Asaia* spp. studied so far. At least 3585 coding sequences were predicted. Insect-associated *Asaia* carried more glycoside hydrolase (GH)-encoding genes than those isolated from plants, showing their high plant biomass-degrading capacity in the insect gut. W12 had the most predicted regulatory protein components comparatively among the selected *Asaia*, indicating its capacity to adapt to frequent environmental changes in the mosquito gut. Two complete operons encoding cytochrome *bo_3_*-type ubiquinol terminal oxidases (*cyoABCD-1* and *cyoABCD-2*) were found in most *Asaia* genomes, possibly offering alternative terminal oxidases and allowing the flexible transition of respiratory pathways. Genes involved in the production of 2,3-butandiol and inositol have been found in *Asaia* sp. W12, possibly contributing to biofilm formation and stress tolerance.

## 1. Introduction

Bacteria of the genus *Asaia* are classified as acetic acid bacteria (AAB) in the class Alphaproteobacteria, family Acetobacteraceae; they are Gram-negative aerobic rods [1]. These bacteria are frequently isolated from tropical plants such as *Bauhinia purpurea* and *Plumbago*, where they metabolize plant sugars and alcohol for growth [2]. Bacteria of the genus *Asaia* have been characterized as symbionts of several insect species, including the orders *Diptera* (flies, including mosquitoes), *Hymenoptera* (bees and wasps), and *Hemiptera* (true bugs), which feed upon plant sugars from the nectar, fruit, and sap [2,3]. Diverse *Asaia* strains have been demonstrated to be tightly associated with several species of *Anopheles* mosquitoes known to be vectors of human malaria (*e.g.*, *A. stephensi*, *A. maculipennis*, and *A. gambiae*) [4]. *Asaia* was one of the most predominant bacterial members found in samples of male and female mosquito midguts [4,5]. Moreover, they persist in host mosquitoes without variations due to sex, blood and sugar meals, and age [5,6]. *Asaia* species live in the mosquito midgut lumen but also actively colonize other tissues and organs, including the salivary glands and reproductive organs, indicating that, if ingested in a sugar meal from a plant, *Asaia* bacteria pass through the tissue body barriers, such as midgut epithelium and basal lamina, reaching and infecting other tissues and organs [1]. Further, the infection of *Asaia* among mosquitoes occurs by horizontal transmission through mating (venereal transmission from adult male to adult female), as well as by vertical transmission from mother to progeny via ovarian infection to eggs [7,8].

The infection of *Asaia* bacteria in insects appears to be of a mutualistic nature, in that the bacteria contribute physiologically to their insect hosts [9]. For example, they provide certain nutrients and metabolic cofactors (such as carbon, nitrogen, and vitamins) [10], positively affect mosquito growth and development, as evidenced by the negative effects upon their removal, and antagonize *Wolbachia* endosymbionts [11,12]. These intimate interactions between *Asaia* bacteria and mosquitoes expand to interference in the development and propagation of *Plasmodium* malaria parasites in *Asaia*-infected *Anopheles* mosquitoes [13], supporting the potential use of these bacteria (in proof of concept) as a paratransgenesis agent to control malaria transmission [1,13,14,15]. The traits favoring this idea are ease of cultivation *in vitro*, amenability for genetic manipulation, and quickly established and stably persistent infections in several mosquito species [1,7,13,14,16]. The strain *Asaia bogorensis* SF2.1 was engineered to secrete anti-plasmodial proteins into the *Anopheles* midgut, resulting in the inhibition of malaria parasite development [16]. Further, introduction of “native” *Asaia* isolates modulated mosquito innate immunity by activation of antimicrobial peptide expression, thereby repressing growth and propagation of parasites in insect guts [17], and more generally appear to compete for space and nutrients with other microorganisms in insect hosts [7,18]. To resolve the issues about mutual exclusion between *Asaia* and *Wolbachia* [14], the latter unrelated bacteria infecting reproductive tissues (and having anti-parasite properties supporting paratransgenesis control methods), and to make use of the favorable traits from the bacteria of both genera, chimeric *Asaia* symbionts expressing a *Wolbachia* surface protein were created that efficiently stimulated mosquito immunity, inhibiting filarial parasite development [15].

Despite their importance for mosquito physiology and the potential for the control of vector-borne disease, the mechanisms involved in establishing symbiosis between *Asaia* and their hosts, regulating the mosquito host development and immunity, remain largely unknown [10,11]. In this study, we isolated a new *Asaia* strain from *A. stephensi*, sequenced the genome, and performed comparative genomic studies with other *Asaia* strains from insects and plants. The goal of this study was to develop a comparative genomic analysis of *Asaia* strains that provides insight into the molecular mechanisms for transmission, colonization, and persistence in mosquitoes. 

## 2. Materials and Methods

### 2.1. Bacterial Strains and Growth Conditions

Adult *A. stephensi* Liston mosquitoes (Johns Hopkins strain) were collected from a laboratory colony maintained at Michigan State University [19], anesthetized for five minutes at −20 °C, and surface-disinfected by soaking in 70% ethanol. Mosquito midgut tissues were dissected under sterile conditions, suspended in 200 µl of sterile saline solution (0.9% NaCl), and homogenized using a pestle. Suspensions were transferred to enrichment broth containing 2.2% D-glucose, 0.5% peptone, and 0.5% yeast extract (pH 3.5). Cultures were rotated at 200 rpm and 30 °C overnight and plated onto a selective medium containing 2.2% D-glucose, 1.0% ethanol, 1.0% yeast extract, 0.7% CaCO_3_, and 1.5% agar. Colonies showing clear zones were isolated and selected for further experiments. Trypticase soy broth (TSB) medium was then used for the culture of *Asaia* isolates resulting from this procedure. One of these isolates, herein designated *Asaia* sp. W12, was chosen for this study because it was predominant. A PCR amplification method was utilized to screen *Asaia* colonies by using the forward primer GCGCGTAGGCGGTTTACAC and reverse primer AGCGTCAGTAATGAGCCAGGTT [1].

### 2.2. Genome Sequencing, Assembly, and Annotation

Isolation and purification of bacterial genomic DNA were performed with the Wizard Genomic DNA Purification Kit (Promega, Madison, WI, USA). Next-generation sequencing (NGS) libraries were prepared using the Illumina TruSeq Nano DNA Library Preparation Kit following the standard procedures recommended by the manufacturer. De novo assembly was performed using CLC NGS Cell v. 10.0.1. Gene annotation was carried out by the NCBI Prokaryotic Genome Automatic Annotation Pipeline (PGAAP 3.3). Genomic data of selected *Asaia* genomes were obtained from the NCBI genome database (https://www.ncbi.nlm.nih.gov, accessed date: 5 January 2020).

### 2.3. Bioinformatics

Functional categorization and classification for predicted ORFs were performed by the RAST server-based SEED viewer [20]. For genome similarity assessment, the average nucleotide identity (ANI) and Digital DNA-DNA Hybridization (dDDH) were computed using GGDC (https://ggdc.dsmz.de, accessed date: 5 January 2020). The pan genome, core genome, and specific genes of *Asaia* spp. were compared to representative *Asaia* genomes using EDGAR 2.0 [21]. The presence of clustered regularly interspaced short palindromic repeats (CRISPRs) in the selected genomes was predicted using CRISPRFinder with the default parameters (https://crispr.i2bc.paris-saclay.fr/Server/, accessed date: 30 January 2020). Among them, questionable CRISPRs were omitted. A cluster analysis of the orthologous groups (COGs) was carried out using Orthovenn2 (https://orthovenn2.bioinfotoolkits.net/task/create, accessed date: 1 April 2021) with an e-value cutoff of 0.001 and an inflation value of 1.5. The taxonomic assignment of *Asaia sp*. W12 was conducted using GTDB-Tk [22] with default settings, which placed this bacterium in the *Asaia* genus. Genomic islands (GIs) were predicted by both IslandPick and IslandPath-DIMOB methods [23].

Eukaryotic-like proteins (ELPs) containing the motifs tetratrico peptide repeats (TPRs: PF13429, PF13371, PF00515, PF13181, PF13432, PF14559, PF14561, and PF09976); ankyrin repeats (ANKs: PF12796 and PF13637); Sel1 repeats (PF08238); and fibronectin type III (PF14310) were predicted using InterProScan v5.44.79 (http://www.ebi.ac.uk/interpro/search/sequence/, accessed date: 5 January 2020). Prediction of the regulatory elements was done using the p2rp program with default settings (http://www.p2rp.org, accessed date: 1 January 2020). Carbohydrate-Active enZyme (CAZyme) families, including enzymes of glycan assembly (glycosyltransferases, GT) and deconstruction (glycoside hydrolases, GH, polysaccharide lyases, PL, and carbohydrate esterases, CE), were semi-manually annotated using the CAZy database curation pipelines [24,25,26]. More precisely, CAZymes were annotated based on a combination of BLASTP and HMMER searches and automatically processed when a high similarity to the reference CAZymes was observed and manually curated in intermediary similarity levels. The genomic context was inspected using GFF files and NCBI genome browsers [26]. The identification of secondary metabolites was performed using the online server antiSMASH 5.0 with “relaxed” detection strictness [27].

### 2.4. Accession of the Genome Sequences

The data from these Whole-Genome Shotgun projects have been deposited at DDBJ/ENA/GenBank under accession number PNQZ00000000.1. The BioProject designation for this project is PRJNA427835, and the BioSample accession number is SAMN08274829.

### 2.5. Statistical Analyses

Statistical analyses were performed using SAS (version 9.2; SAS Institute, Cary, NC, USA).

## 3. Results

### 3.1. Genome Features

**Figure 1:**
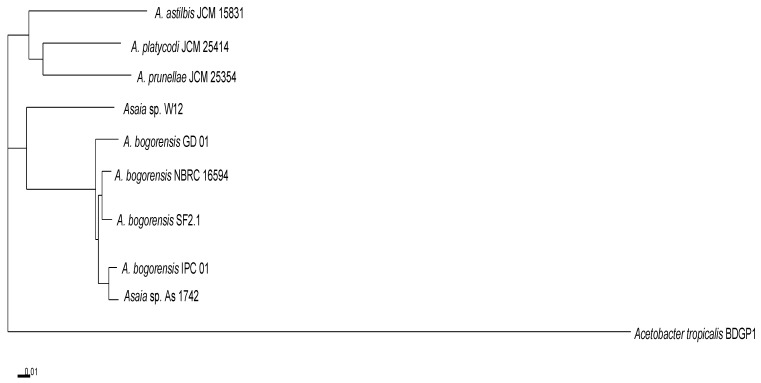
Phylogenetic placement of the selected *Asaia*. The tree is calculated from 829 core amino acids sequences per genome (6632 core amino acid sequences). The tree showed 100% branch support in 250 bootstrap iterations. The tree for 10 genomes was build out of a core of 471 genes per genome, which was 5181 in total. The selected genomes were: *Acetobacter tropicalis* strain BDGP1 (CP022699) (outgroup), *A. astilbis* JCM15831 (BAJT01000001), *A. bogorensis* NBRC16594 (AP014690), *A. bogorensis* GD01 (UEGO00000000.1), *A. bogorensis* IPC01 (UBIX00000000.1), *A. platycodi* JM 25414 (BAKW01000001), *A. prunellae* JCM 25354 (BAJV01000001), *Asaia* sp. As1742 (VWWA00000000), *A. bogorensis* SF2.1 (CBLX010000009.1), and *Asaia* sp. W12 (PNQZ00000000.1).

**Figure 2:**
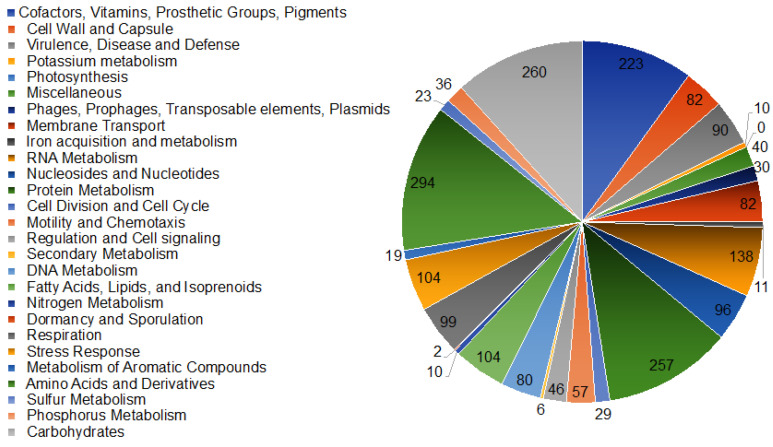
Subsystem category distribution of *Asaia* using SEED subsystems by the RAST analysis. The pie chart represents the relative abundance of each subsystem category, and the numbers depict the subsystem feature counts.

A GTDB-Tk analysis placed the bacterium W12 in the genus *Asaia* [22]. *Asaia* sp. W12 formed a clade with mosquito-associated *A. bogorensis* SF2.1 and *A. bogorensis* GD01, as well as ant-associated *Asaia* As-1742 (Figure 1); however, it departed from the cluster formed by plant-associated *A. prunellae* JCM25354, *A. platycodi* JCM25414, and *A. astilbis* JCM15831 (Figure 1). Overall, the *Asaia* species showed a different evolution pathway from the outgroup representative *Acetobacter tropicalis* strain BDGP1 (Figure 1). The genome sizes of the insect-associated *A. bogorensis* (i.e., *A. bogorensis* SF2.1 and *A. bogorensis* GD 01) were more similar to those isolated from the plants (Table 1). *Asaia* sp. W12 had the largest genome size (3.94 M) among the selected *Asaia* (Table 1). However, the average genome sizes (Table 1) in the insect-associated *Asaia* species (average 3.59 M, *n* = 4) were significantly larger (*t*-test, *p* < 0.05) than those from plant-associated ones (average 3.17 M, *n* = 5). The assembled genome of *Asaia* sp. W12 contained 229 contigs and 3652 coding sequences (CDSs) (Table 1). The average GC content in W12 was 60.1%, consistent with those in most of the selected *Asaia* species. It is interesting that the average GC content in *Asaia prunellae* JCM 25354 was much lower (55.8%) than that in the other *Asaia* (Table 1). A genome analysis of *Asaia sp.* W12 by the RAST Server revealed at least 378 subsystems classified into 27 categories (Figure 2). Among these categories, the “amino acid and derivatives” subsystem had the largest number (294 CDSs), followed by carbohydrate metabolism (260), protein metabolism (257), and RNA metabolism (138). Moreover, the “stress response” category accounted for at least 104 CDSs. Within the “virulence, disease, and defense” subsystem (90 CDSs), 12 of them were related to invasion and intracellular resistance, while 72 were associated with a resistance to antibiotics and toxic compounds (Figure 2).

### 3.2. Gene Repertoire of Asaia *spp.*

**Figure 3:**
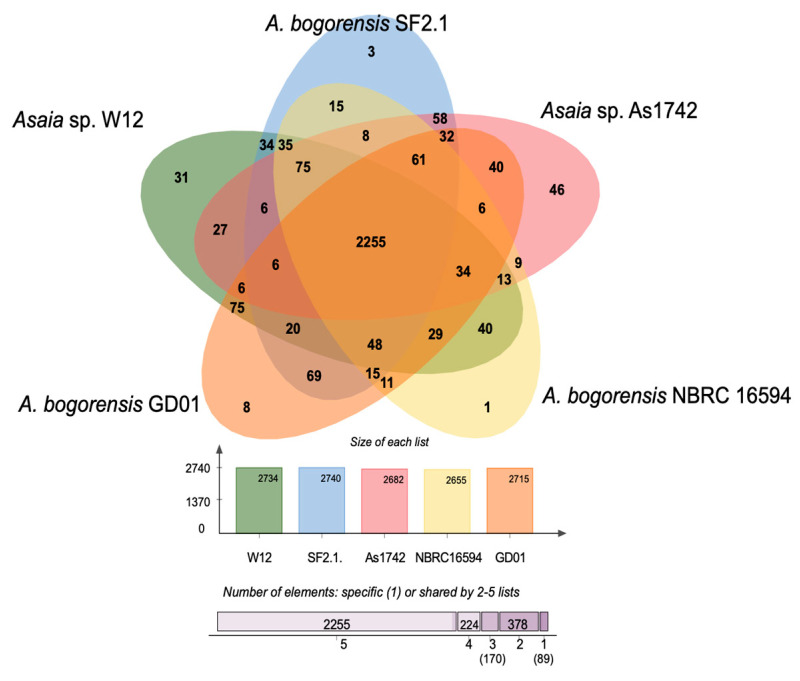
Proteome comparison among the selected Asaia. Venn diagrams generated by OrthoVenn 2.0 showing orthologous clusters shared separately by *Asaia* sp. W12 (green) and four other close *Asaia* species—specifically, *A. bogorensis* SF2.1 (blue), *Asaia* sp. As1742 (pink), *A. bogorensis* NBRC 16594 (yellow), and *A. bogorensis* GD01 (orange). When *Asaia* sp. W22 was compared with the other *Asaia*, there were 378, 170, 224, and 2255 clusters shared between 2, 3, 4, and 5 strains, respectively, while 89 clusters (clusters of singleton genes) were found in only 1 of the 5 *Asaia* compared.

A genome-wide comparison of the orthologous clusters in different isolates provides insight into the gene structure, gene function, and molecular evolution of genomes (Figure 3). The species form 3116 clusters, 912 orthologous clusters (at least contains two species), and 2204 single-copy gene clusters. The COG analysis of *Asaia* sp. W12 was compared with the other four selected genomes (Figure 3). The analysis shows that *Asaia* sp. W12 contained 2734 COGs. Among them, 2256 COGs were shared by all five strains, and 31 COGs were only present in the *Asaia* sp. W12 genome (Figure 3). There are only three and eight COGs present in mosquito-associated *A. bogorensis* SF2.1 and *A. bogorensis* GD01, respectively. The unique COGs existing in *Asaia sp.* W12 involved several genes functioning with amino acid transport, regulation of the amino acid catabolic process, urea metabolic process, fatty acid biosynthetic process, DNA-binding transcription factor activity, and many others. However, the representative meanings of these singular genes in W12 are not clear. Further investigations to understand the features of these unique genes in W12 are warranted. To investigate the pangenome in *Asaia*, we plotted the number of annotated genes, shared genes, and unique genes as a function of the number of sequenced genomes (Figure S1).

### 3.3. Carbohydrate-Active EnZymes (CAZymes) in Asaia *spp.*

**Figure 4:**
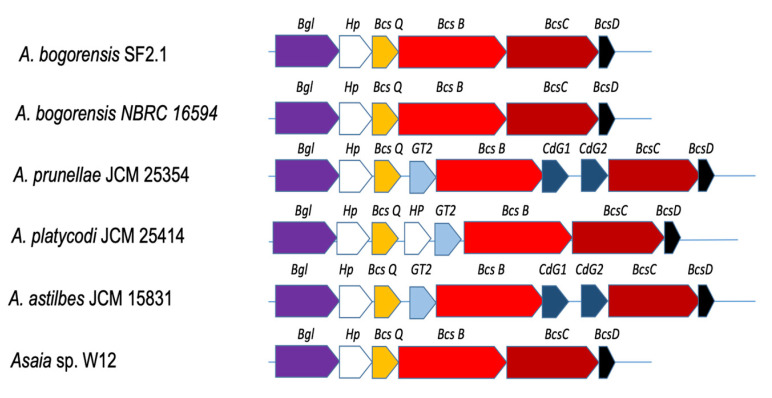
The operon organization of bacterial cellulose biosynthesis in selected *Asaia* strains. *Bgl* (purple); β-glucosidase gene; *Hp* (white), hypothetical protein gene; *BcsQ* (yellow), bacterial cellulose synthase (BCS) Q gene, involved in cellular localization of the BCS complexes; *BcsB* (red)*,* bacterial cellulose synthase subunit B gene; *BcsC* (dark red)*,* bacterial cellulose synthase subunit C gene; *BcsD* (black)*,* bacterial cellulose synthase subunit D gene; *GT2* (blue)*,* glycosyltransferase family 2 gene; *CdG*, C-di-GMP precursor gene (dark blue).

The number of glycoside hydrolase (GH) genes in insect-associated *Asaia* (i.e., W12, SF 2.1, As-1742, and *A. bogorensis* GD-01) was slightly higher than those originating from plants (Table 2). Gene copies of glycosyltransferases (GTs) or carbohydrate esterases (CEs) in *A. astilbis* JCM 15831 and *A. platycodi* JCM 25414 were lower than those from other selected *Asaia* (Table 2). Most CAZymes in *Asaia* genomes (~30%) targeted peptidoglycan (glycoside hydrolases families GH23, GH102, GH103, GH104, and GH108), while we also observed the conservation of some CAZymes dedicated to sucrose or fructose polymers (GH32 and GH68) that could, for example, allow the metabolism of plant nectar by mosquitoes (Table S1). Interestingly, these genomes also encode a trehalase (GH37) commonly found in various organisms (Table S1). Trehalose plays a critical role as an energy source in insects, and it is involved in tolerating abiotic stresses [28]. The bacterial trehalase may contribute to the host carbon metabolism process and defense against osmotic and oxidative stress [29]. Despite the variable genome sizes and assembly across these genomes, two relevant CAZyme operons conserved in most species were identified. The first operon includes the bacterial glycogen operon, with α-glucanases (with four GH13s from distinct subfamilies for linkage specificities, three appended to the carbohydrate-binding module family CBM48) and glycosyltransferases, notably from the GT4 and GT5 families, in a single locus (Table S1). The second one included a GH8 and a glycosyltransferase of the GT2 family likely involved in a cellulose-like biofilm synthesis (Table S1). The assembly of the bacterial cellulose biosynthesis operons was diverse among the *Asaia* species (Figure 4).

### 3.4. Regulatory Systems in Asaia *spp.*

The *Asaia* sp. W12 genome encoded 57 two-component system proteins, 169 transcription factor proteins, and 17 other DNA-binding proteins, which possessed the most regulatory protein genes (total 243) among the selected *Asaia* (Table 3). Next to it, *Asaia* sp. As-1742 (ant-associated) carried at least 235 predicted regulatory genes encoding 46 two-component system proteins, 174 transcription factor proteins, and 15 other DNA-binding proteins. Similar to those in the two insect isolates, the mosquito isolate *A. bogorensis* SF2.1 also had abundant regulatory proteins (up to 213 regulatory protein genes and components). Remarkably, most plant-associated strains possessed relatively fewer regulatory proteins (ranging from 150 to 198) and components (Table 3). For example, the total number of transcriptional regulators (TRs, 81) and one-component systems (OCSs, ranging from 40 to 57) in these insect isolates was relatively higher than those in the plant-associated ones (Table 3). However, regardless of their origin, *Asaia* bacteria have comparable numbers of RRs, phosphotransferase proteins (PPs), and sigma factors (SFs) in their genomes, indicating that these regulatory proteins play the fundamental roles in maintaining bacterial metabolism and function.

### 3.5. Genes Involved in other Symbiosis Traits

We further investigated additional well-known genetic traits possibly important for forming a symbiotic relationship with its host mosquitoes (Table 4). For example, W12 and other selected *Asaia* (except *A. astilbis* JCM 15831) carried two complete *cyoABCD* operons encoding bo3-type ubiquinol oxidase genes (Table 4). The complete operons of *cyoABCD* encoded bo3-type ubiquinol oxidases with four subunits [30]. The presence of the additional copy of *cyoABCD* operons may contribute to survival in the dramatically different environmental changes in hosts (such as gut vs. saliva). Volatile compounds (BVCs) such as 2,3-butanediol (2,3-BD) and acetoin affect biofilm formation, bacterial motility, and associations with hosts [31,32]. The putative acetoin and butanediol synthesis pathways were discovered in all the selected *Asaia* genomes, indicating BVCs may contribute to the formation of a symbiosis relationship (such as colonization) between *Asaia* and their plant or insect hosts. Inositol is an important nutritional or signaling factor in many microbes and eukaryotes [33]. The inositol metabolic pathways found in *Asaia* may participate in the regulation of the stress response (such as cold tolerance) in hosts [34]. Moreover, *Asaia* had operons involved in flagella formation, which accounted for the ability to migrate to several locations in mosquitoes [1]. A gene encoding a large adhesin (filamentous hemagglutinin family outer membrane protein) possibly functioning in the attachment to host cells [35] was found in all but *A. bogorensis* SF2.1. Several motifs participating in protein–protein interactions were detected in members of the genus *Asaia* (Table 5). The protein-coding genes containing the eukaryotic-like motifs/eukaryotic-like proteins (ELPs) with TPRs (7~10 per genome), ANKs (1 to 2 per genome), Sel1-like repeats (2 to 3 per genome), and Fn3 (1~3 per genome) showed that *Asaia* bacteria had many different ways to adhere to the cell surface and invade the host tissues.

### 3.6. Comparative Genome Plasticity

Among the selected *Asaia* spp., only *A. bogorensis* NBRC 16594, *A. astilbis* JCM 15831, and *Asaia* sp. W12 carried intact prophages (Table S2). At least seven different prophages were predicted in the genome of *Asaia* sp. W12, including one intact (prophage IV), one questionable (prophage VI), and five incomplete ones (Table S2). The largest one (prophage IV, 30.6 Kb) contained a battery of genes encoding the phage tail, head, portal, integrase, lysin, terminase, and other component proteins. Prophage VI (23.4 Kb) carried at least 35 CDS encoding the ApaG protein, O-succinylhomoserine sulfhydrylase, DNA polymerases, primase/helicase proteins, exonuclease, lysozyme, several hypothetical proteins, and phage structural and assembly proteins. The other predicted prophages (sizes ranging from 9.0 to 19.8 Kb) seemed to be incomplete, because they lacked the full set of structural or assembly elements. However, these “incomplete prophages” may utilize elements from complete ones. Some of the incomplete prophages may be involved in fitness and adaption under certain conditions. For example, the third-largest one (prophage I, 19.8 Kb, incomplete) contained transcriptional regulators, the siderophore interacting protein, stress response protein, and several phage structure proteins (Table S2). Prophage III (19.7 Kb, incomplete) carried multiple transposases, killer proteins (HigA and HigB), and hydrolases. Moreover, prophage IV may have integrated into the host genome long ago, because its GC content (61.5%) is only slightly different from average in the whole genome (60.1%). Remarkably, the GC content of two complete prophages in *A. astilbis* JCM 15831 (56.0% and 56.3% in prophage I and III, respectively) was much lower than average in the genome, indicating that they are recently invaded phage elements. Similarly, a lower GC content (57.8%) of the intact prophage II predicted in *A. bogorensis* NBRC 16594 was found. Interestingly, *A. bogorensis* SF2.1 associated with mosquitoes did not carry any complete prophages, highlighting the differential evolution of these *Asaia* species and strains (Table S2). 

Up to 17 genomic islands (GIs) were detected in the *Asaia* sp. W12 genome, ranging in size from 4.05 to 72.04 Kb (Table S3). Genes encoding flagellar structural and assembly proteins; prophage components; various enzymes (e.g., lipase, proteases, lysozyme, NAD(P)H oxidoreductase, hydrolases, and amidase); DNA metabolism; transposases; regulators; modification and restriction systems; and stress response systems occurred in these GIs (Table S3), indicating that *Asaia* sp. W12 possibly acquired these genes, thereby forming GIs favoring the adaptation to diverse environments. 

## 4. Discussion

One of the striking features in insect-associated *Asaia* species is that they have much larger genome sizes than plant-associated ones, which is consistent with that reported by Comandatore et al. [36]. This observation highlights the diverse lifestyles of *Asaia* species forming a symbiotic relationship with mosquito hosts [1]. Mosquitoes live in both aquatic and terrestrial environments (depending on their developmental stages) and need various diets (microorganisms and detritus as larval food; sugar and blood as adults) [13]. Such frequent and dramatic changes necessitate mosquito-associated *Asaia* to carry out versatile metabolic activities and regulatory mechanisms (see below). *Asaia* species experienced an evolution that occurred through independent genomic reduction/variation after they formed symbiotic relationships with mosquitoes [36,37]. For example, some genes were reduced in COG pathways, including those involved in transcription, replication, recombination, and repair, as well as cell motility [36].

Differences in the repertoires of these regulatory proteins are likely to facilitate the adaptation of *Asaia* to different hosts and/or could be responsible for different symbiosis or disease characteristics induced [2,5,16]. Strain W12 carried the most regulatory protein genes among the selected genomes, which may indicate a high degree of adaptability of this organism to both plant and insect environments [38]. *A. bogorensis* SF2.1 was reported to be located in multiple sites in mosquitoes, including the salivary gland, midgut, and reproduction organs [13]. These niches obviously represent very different microenvironments physiologically [2,37]. One possible explanation is that these signaling molecules trigger the expression of genes responsible for stress and regulate the development of the symbiosis relationship or intercellular communication [10,12,39]. Several signaling proteins are present in insect-associated Asaia while commonly absent in plant-associated *Asaia*. The acquisition of transcriptional protein genes in insect-associated *Asaia* cannot be explained by genome size increases, because the relative transcriptional protein ratio was also higher (average 61/Mb) than those from insects (average 53/Mb). During the evolution of various symbiotic relationships with different hosts, bacteria tend to maintain their target genes more than the transcription factors that regulate their expression [40]. Our present results indicated that mosquito-associated *Asaia* obtain/maintain more transcription factors to control their gene expression in order to adapt to the complex environment, which may stress the strain difference and various evolution pathways. However, the challenge remains to associate these differences in TCS proteins to specific traits of *Asaia*.

Most of the CAZymes in *Asaia* species are involved in metabolizing simple sugars rather than complex plant polysaccharides [41], which is consistent with their living conditions [5]. The increased number of glycoside hydrolases in insect-associated *Asaia* strains were only limited to a few peptidoglycan lyases or chitinase-like hydrolases, showing that the adaptation to a plant or insect host was not marked by the acquisition of specific CAZymes [2,41]. It has been well-documented that *Asaia* and other acetic acid bacteria produce bacterial cellulose (BC), which contributes to cell adherence to plant surfaces or insect epitheliums or other tissues, as one of the biofilm components [42]. The physiological role of BC may be important for the symbiotic relationship persistence between *Asaia* and mosquitoes by (1) hindering the flagellar rotation, (2) limiting cell motility, and (3) promoting biofilm formation [42]. The mechanisms involved in bacterial cellulose formation and its regulation in *Asaia* are not clear; our findings suggest that bacterial cellulose biosynthesis is diverse among *Asaia* strains, implying that the regulatory mechanisms of BC-related biofilm are different. It is well-known that the cellulose matrix extracted from *Asaia* has thinner fibrils, highlighting the biological origination differences from those well-known bacteria [43]. The cellulose-producing bacterium *Gluconacetobacter xylinus* has endo-1,4-β-glucanase and β-glucosidase genes (located adjacent to the cellulose synthesis operon), which played an important role in regulation of cellulose biosynthesis [44]. However, β-glucosidase genes in *A. bogorensis* were outside of the cellulose synthesis cluster, which produced a different type of β-glucosidases [45]. The different cellulose productivities between *A. bogorensis* and *G. xylinus* may partly be linked to the different β-1,4-glucanases [45]. *bscA* was not present in this operon, as were the typical ones discovered in *E. coli* and others [46]. The second gene in the operon (*bcsB*) encodes the catalytic subunit of cellulose synthase [47]. Bacterial strains mutated in the *bcsA* locus were found to be deficient in cellulose synthesis due to the lack of cellulose synthase and diguanylate cyclase activities [47].

Besides the above-mentioned traits, we also further investigated eukaryote-like proteins (ELPs) in *Asaia* genomes, which may lead to the discovery of novel mechanisms underlying host–symbiont interactions [48]. These domains may be acquired through horizontal gene transfer (HGT) from the eukaryote hosts or through convergent evolution [49]. ELPs have only been reported in a few symbiotic bacteria in mosquitoes [50,51,52]. However, they are likely to be more widespread. *Asaia* species carried several genes encoding ELPs, including TPR, Ank, Sel1 repeats, and Fn3. ELPs in the mosquito symbiont *Wolbachia pipientis w*Mel carried at least 23 ANK-containing genes [51,52], indicating that the contents of the ELPs were beyond the strict pathogens. Further, Klasson et al. (2008) reported that *W. pipientis w*Pip had up to 60 ANK proteins with some of unique presence in its own genome [53]. *Cardinium hertigii* (another inherited bacterial symbionts causing CI) was reported to have many ELPs (such as ANK-containing proteins) [54].

Even though the bacterial niches in mosquito tissues and organs are not strictly anaerobic, some tissues may have limited oxygen concentrations. Coon et al. (2017) showed that bacteria (wild-type *E. coli*) reduced the midgut oxygen concentrations below 5% in both nonsterile or gnotobiotic larvae [55]. However, *E. coli* mutants lacking cytochrome *bd* oxidase genes did not [55]. In the same study, they further demonstrated that hypoxia mediated by bacterial respiratory functioned as an important signal of larval development and ecdysone-induced molting [55]. Together, their findings indicated the importance of aerobic respiration by gut bacteria in mosquito development. Here, our comparative genome analysis showed that, regardless of the species’ origination, most *Asaia* genomes carried two distinct *cyo* operons (except *A. astilbis* JCM 15831). Previously, Chouaia et al. (2014) found that only eight AAB species (animal and plant pathogens or symbionts, including *A. bogorensis* NBRC 16594) among the 705 bacterial genomes contained two complete *cyo* operons [38]. The expression level of the two *cyo* operons was differently regulated, depending on their culture conditions [38]. One can infer that the additional copy of fully functional modules of *bo3* and *bd* may allow bacteria *Asaia* to handle oxidative stress conditions in mosquitoes. They may actively create hypoxia (a signal for molting), a condition in the mosquito gut, using their enhanced respiratory capability [55]. It will be necessary to test which *cyo* operon(s) directly participate in the above process and which may serve to create tools for interrupting growth of larval mosquitoes. 

In many microbes, pyruvates can be channeled via α-acetolactate and acetoin into 2,3-butanediol. The production of this compound is induced under limited oxygen levels and low pH, which possibly happens in some mosquito tissues [56,57]. *P. aeruginosa* grown with the 2,3-butanediol supplement persisted longer in the murine respiratory tract [58]. They promoted TNF-α and IL-6 responses in the host and led to the increased colonization of the microbiota in the lung tissue [58]. Further, 2,3-butanediol upregulated the expression of the global transcription regulator *LasI-LasR* controlling the quorum sensing in *P. aeruginosa*, which increased the exotoxin concentrations and biofilm formation [59]. Due to the importance of 2,3-butanediol in biofilm formation and toxicity [59], one can hypothesize that it not only has a role in bacterial antagonism and *Asaia* abundance but, also, in the increased colonization of the mosquito midgut with other environmental microbes. It is also worth noting that inositol metabolic pathways were found in all of the selected *Asaia*. Pathogenic bacteria (such as *Mycobacterium*) require inositol biosynthesis for survival and pathogenesis, because inositol may be involved in detoxification and in protecting the cell from oxidative damage in the course of infection [60]. In animals, myo-inositol participated in several cellular signaling pathways and functioned as an osmolyte in some specific tissues where osmolarity played a crucial biological role [61]. For example, the ability of insects to survive during cold environments (such as a harsh winter) possibly links to myo-inositol production [62]. Therefore, it is possible that inositol metabolic pathways in *Asaia* contribute to the stress response regulation (such as cold tolerance).

Horizontal gene transfer (HGT), transposons, and prophages often lead to genome plasticity [63,64]. Among them, prophages are very important for genetic diversification by delivering functional genes among different strains [65,66]. For example, some transcriptional regulators, iron uptake proteins, stress response proteins, killer proteins (HigA and HigB), and hydrolases were found in the prophages of *Asaia*. The size of the genome and the numbers and size of the GIs in W12 were more than those in other selected *Asaia*, implying that W12 had a more flexible response to environment changes. The flagellar structural and assembly protein genes; various enzymes (e.g., lipase, proteases, lysozyme, NAD(P)H oxidoreductase, hydrolases, and amidase); DNA metabolism; transposases; modification and restriction systems; and stress response systems were found in these GIs. *Asaia* bacteria possibly employ a complex set of chemosensory pathways to swim towards different insect organs and adapt to the insect’s physiological demands. However, the detailed mechanisms need to be further investigated.

## 5. Conclusions

In conclusion, *Asaia* sp. W22 carried several genetic traits that facilitate the formation of symbiotic relations with insects [10,18,37]. It had more signaling components and glycoside hydrolase genes compared to many other selected *Asaia* genomes. Many well-known eukaryotic-like motifs/eukaryotic-like proteins (ELPs) and large adhesins involved in the protein–protein interactions for a range of cellular processes were discovered in its genome. Remarkably, most *Asaia* species carried two copies of *cyoABCD*, encoding *bo3*-type ubiquinol oxidase genes (encoding the terminal respiratory chain protein), which was a unique characteristic for them to be symbionts in both plants and insects [38]. Metabolic genes, including inositol and butanediol synthesis, possibly play the important roles in stress tolerance and biofilm formation.

## Figures and Tables

**Table 1:** General features of the various *Asaia* spp.

Species	Sources	Size (Mb)	GC%	Total RNA Number *	CDS	GenBank Accession Number
*A. bogorensis* NBRC 16594	*Bauhinia purpurea*	3.20	59.8	59	2896	GCA_001547995.1
*A. astilbis* JCM 15831	*Astilbe thunbergii*	3.15	58.0	49	3482	GCA_000613845.1
*A. prunellae* JCM 25354	*Prunella vulgaris*	3.18	55.8	48	3583	GCA_900465315.1
*A. platycodi* JCM 25414	*Platycodon grandiflorum*	3.15	59.3	49	3774	GCA_000614545.1
*A. bogorensis* IPC-01	Flower	3.17	59.7	54	2880	GCA_900465315.1
*A. bogorensis* GD01	*Anopheles gambiae*	3.34	59.8	60	3226	GCA_900465345.1
*A. bogorensis* SF2.1	*Anopheles stephensi*	3.52	59.8	45	3005	GCA_000724025.1
*Asaia* sp. As-1742	*Atta sexdens*	3.74	59.6	54	3308	GCA_011753235.1
*Asaia* sp. W12	*A. stephensi*	3.94	60.1	59	3580	GCA_003994335.1

* The total RNA number includes the rRNA and tRNA copies.

**Table 2:** The distribution of glycoside hydrolases, glycosyltransferases, and carbohydrate esterases amongst different *Asaia* spp.

	Glycoside Hydrolases (GHs)	Glycosyltransferases (GTs)	Carbohydrate Esterases (CEs)
*A. astilbis* JCM 15831	17	37	1
*A. platycodi* JCM 25414	15	34	1
*A. prunellae* JCM 25354	22	55	1
*A. bogorensis* NBRC 16594	28	54	3
*A. bogorensis* IPC-01	27	54	3
*A. bogorensis* GD-01	28	55	3
*A. bogorensis* SF2.1	32	53	3
*Asaia* sp. As-1742	32	53	3
*Asaia* sp. W12	32	52	3

**Table 3:** Regulatory systems in *Asaia* spp.

	Predicted Regulatory Proteins								
	Two Component Systems			Transcription Factors				Other DNA-Binding Proteins	Total
	RR	PP	HK	OCS	RR	TR	SF	ODP	
*A. astilbis* JCM 15831	19	1	15	27	10	65	5	8	150
*A. bogorensis* NBRC 16594	25	0	22	42	15	62	7	11	184
*A. bogorensis* IPC-01	24	0	20	35	14	64	7	13	177
*A. prunellae* JCM 25354	23	2	14	30	13	69	7	12	170
*A. platycodi* JCM 25414	16	2	16	33	13	59	8	10	157
*A. bogorensis* GD-01	21	1	19	39	13	83	7	15	198
*A. bogorensis* SF2.1	25	1	21	40	15	88	7	16	213
*Asaia* sp. As-1742	24	1	21	57	14	96	7	15	235
*Asaia* sp. W12	30	1	26	57	18	85	9	17	243

Abbreviations: HK, histidine kinases; RR, response regulators; PR, phosphotransferase proteins; TR, transcriptional regulators; OCS, one-component systems; SF, sigma factors; ODP, other DNA-binding proteins.

**Table 4:** Genes involved in other symbiotic traits in *Asaia.*

Species	*cyoABCD*	Acetoin, Butanediol and Inositol	Flagella	Large Adhesin
*A. astilbis* JCM 15831	1	+	+	+
*A. bogorensis* NBRC 16594	2	+	+	+
*A. bogorensis* IPC-01	2	+	+	+
*A. prunellae* JCM 25354	2	+	+	+
*A. platycodi* JCM 25414	2	+	+	+
*A. bogorensis* GD-01	2	+	+	+
*Asaia* sp. As-1742	2	+	+	+
*A. bogorensis* SF2.1	2	+	+	-
*Asaia* sp. W12	2	+	+	+

**Table 5:** Possible eukaryotic-like protein (ELP) motifs in the selected *Asaia* species.

	Tetratrico Peptide Repeats	Ankyrin Repeats	Sel1 Repeats	Fibronectin Type III	Total Motif
*A. astilbis* JCM 15831	9	2	2	2	15
*A. bogorensis* NBRC 16594	9	1	3	1	14
*A. bogorensis* IPC 01	9	1	3	1	14
*A. platycodi* JCM 25414	7	1	3	1	12
*A. prunellae* JCM 25354	9	1	3	3	16
*A. bogorensis* GD 01	8	1	3	1	13
*A. bogorensis* SF2.1	9	1	3	2	15
*Asaia* As-1742	8	1	3	1	13
*Asaia* sp. W12	10	1	2	1	14

## Data Availability

All data generated or analyzed during this study are available from the corresponding author upon reasonable request.

## Supplementary Materials

The following are available online at https://www.mdpi.com/2073-4425/12/5/752/s1, Table S1: The CAZy prediction amongst different *Asaia* spp. Table S2: The putative prophages predicted in various *Asaia*. Table S3: The total genomic islands predicted in various *Asaia* spp. Figure S1: Core genome, pan genome, and singleton genome evolution.
