# Supplementary material for: Genome Features of Asaia sp. W12 Isolated from the Mosquito Anopheles stephensi Reveal Symbiotic Traits
Source: Genes (Basel). 2021 May 17;12(5):752. doi: 10.3390/genes12050752 (PMC8156966; doi:10.3390/genes12050752)
Supplement: Supplementary file 1 [file genes-12-00752-s001.zip › genes-1104148-supplementary/Figure S1.pdf]

A

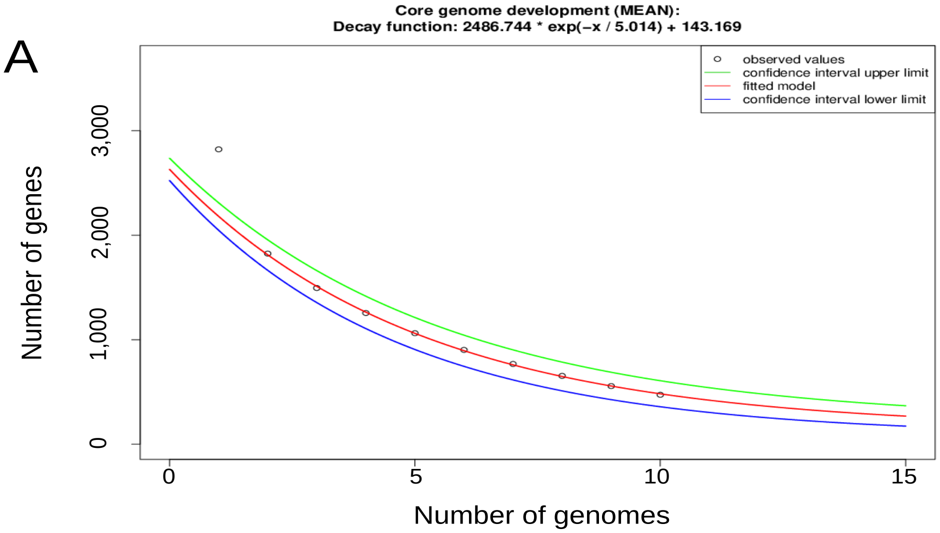
Core genome development (MEAN):
Decay function: 2486.744 * exp(−x / 5.014) + 143.169
observed values
confidence interval upper limit
fitted model
confidence interval lower limit
Number of genes
0
1,000
2,000
3,000
0
5
10
15
Number of genomes

B

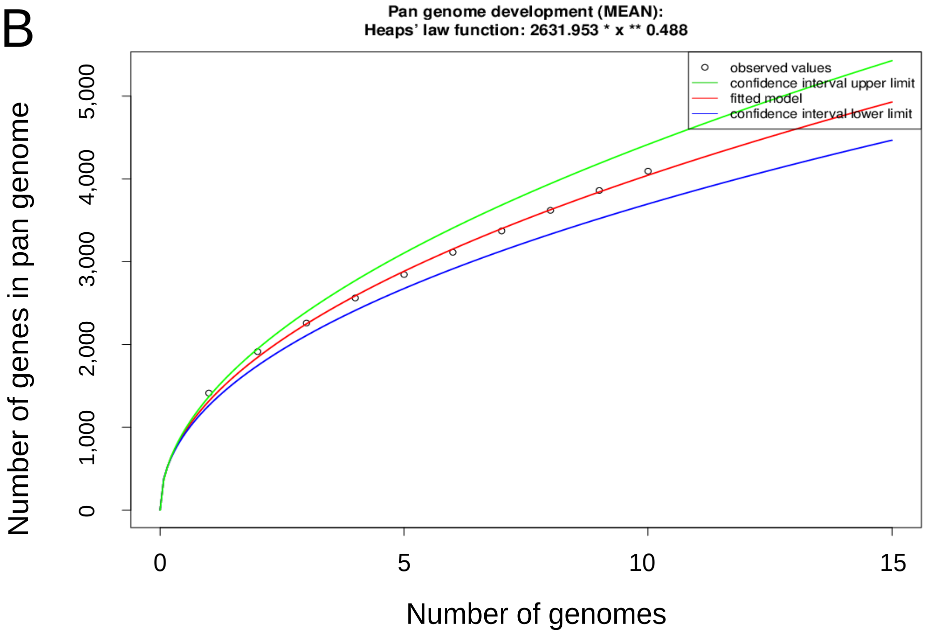
Pan genome development (MEAN):
Heaps' law function: 2631.953 * x ** 0.488
observed values
confidence interval upper limit
fitted model
confidence interval lower limit
Number of genes in pan genome
0
1,000
2,000
3,000
4,000
5,000
0
5
10
15
Number of genomes

C

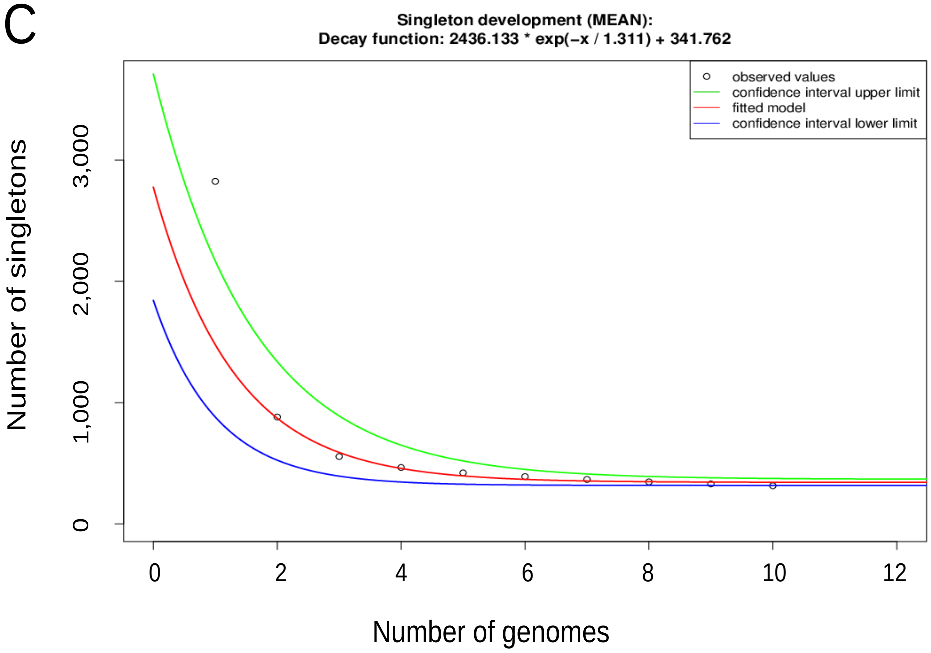

Figure 1. Core genome, pan genome and singleton genome evolution. **A**) Total number of genes (pan-genome) for a given number of genomes sequentially added. **B**) Number of shared genes (core genome) as a function of the number of genomes sequentially added. **C**) Number of unique genes (singleton genome) as a function of the number of genomes sequentially added.
